# On the inverse problem in optical coherence tomography

**DOI:** 10.1038/s41598-023-28366-w

**Published:** 2023-01-27

**Authors:** Callum M. Macdonald, Simon R. Arridge, Peter R. T. Munro

**Affiliations:** 1 Department of Medical Physics and Biomedical Engineering, University College London, Malet Place, Gower Street, London, WC1E 6BT UK; 2 Department of Computer Science, University College London, Gower Street, London, WC1E 6BT UK

**Keywords:** Imaging and sensing, Interference microscopy

## Abstract

We examine the inverse problem of retrieving sample refractive index information in the context of optical coherence tomography. Using two separate approaches, we discuss the limitations of the inverse problem which lead to it being ill-posed, primarily as a consequence of the limited viewing angles available in the reflection geometry. This is first considered from the theoretical point of view of diffraction tomography under a weak scattering approximation. We then investigate the full non-linear inverse problem using a variational approach. This presents another illustration of the non-uniqueness of the solution, and shows that even the non-linear (strongly scattering) scenario suffers a similar fate as the linear problem, with the observable spatial Fourier components of the sample occupying a limited support. Through examples we demonstrate how the solutions to the inverse problem compare when using the variational and diffraction-tomography approaches.

## Introduction

Optical Coherence Tomography (OCT) is an imaging technique based on low-coherence interferometry. It is widely applied as a qualitative imaging modality in ophthalmology where volumetric images provide structural and morphological information of the human eye, with resolutions in the range of a few micrometers^1–4^. Such images are, however, perturbed by speckle and sample-induced aberration. In some cases, such as deep within tissue or in strongly scattering media, image degradation becomes more significant due to phenomena such as multiple scattering. Furthermore, the signal to noise ratio rapidly reduces as a function of imaging depth. Methods to counter these effects have been investigated, such as speckle reduction using spatial and spectral averaging^5^, and aberration correction using adaptive optics^6^. Additionally, various computed imaging techniques have been explored which attempt to improve image quality in locations outside of the focal region of the optics, as well as correcting for aberrations^7–9^.

Whereas these suggested extensions improve image quality they do not address the quantification of optical parameters such as permittivity or conductivity. In this paper we first review the feasibility of optical parameter recovery from OCT data from a theoretical standpoint under a weak scattering approximation. We then consider an inverse problem framework, for both linear and nonlinear scenarios (both weak and strong scattering). This is enabled by the use of a Pseudo Spectral Time Domain (PSTD) solver for propagating the incident and scattered electromagnetic fields^10^.

## Background

### OCT inverse problem

**Figure 1:**
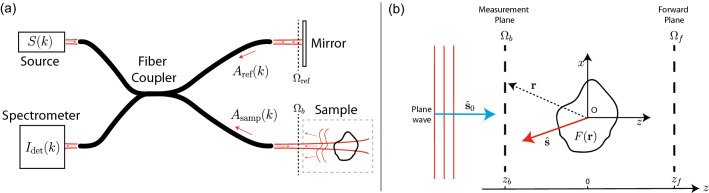
(**a**) A fiber-based Fourier domain OCT interferometer system. *S*(*k*) is the source spectral density. $A_{\text {ref}}(k)$ is the complex valued coupling coefficient of light reflected from sample arm. $A_{\text {samp}}(k)$ is the complex valued coupling coefficient of light reflected from sample arm. $I_{\text {det}}(k)$ is the detected intensity of the combined sample and reference arms of the interferometer. (**b**) Close-up geometry of the scattered field inverse problem. A plane wave travelling in the ${{\textbf {s}}}_{0}$ direction is incident on an object with scattering potential $F({{\textbf {r}}})$. The scattered field ${{\textbf {E}}}_{\text {scat}} ({{\textbf {r}}})$ is measured on a plane normal to the *z*-direction.

OCT employs a broadband light source and images are formed by exploiting the interference between light back-scattered by the sample, and light reflected by a reference mirror. Figure 1a shows the basic system design for fiber-based Fourier-domain OCT, where single-mode fibers are used to direct light within the interferometer, and a spectrometer analyses the intensity signal arising from interference between light from the sample and reference arms. This combined signal, $I_{\text {det}}$, can be expressed in terms of the complex fiber coupling coefficients for the electric field within each arm of the interferometer.

1 $$\begin{aligned} I_{\text {det}}(k) = \vert A_{\text {ref}}(k) \vert ^{2} + \vert A_{\text {samp}}(k) \vert ^{2} + \underbrace{2\,\textrm{Re}\left\{ A_{\text {ref}}^{*}(k) A_{\text {samp}}(k) \right\} }_{\text {Inteference Term}} \;\;, \end{aligned}$$

where $A_{\text {ref}}$ is the coupling coefficient of the reference arm light, $A_{\text {samp}}$ is the coupling coefficient of the sample arm light, *k* is the wavenumber, and ${\textrm{Re}}\{\}$ denotes the real part. OCT depth scans (A-scans) are generated by Fourier transforming $I_{\text {det}}$, resulting in a signal that is approximately proportional to the reflectivity of the sample as a function of depth. Specifically, the component of $I_{\text {det}}$ which contains information relevant to the OCT image is the interference term (3rd term on the right of Eq. 1). To relate the detected signal to the fields present within the sample and reference mirror regions, the coupling coefficients of each arm can be expressed as:

2 $$\begin{aligned} {}&A_{\text {samp}}(k) = \int _{\Omega _{b}} \varvec{\phi }({{\textbf {r}}},k) \cdot {{\textbf {E}}}_{\text {scat}} ({{\textbf {r}}},k)\, d{{\textbf {r}}},\\&A_{\text {ref}}(k) = \int _{\Omega _{\text {ref}}} \varvec{\phi }({{\textbf {r}}},k)\cdot {{\textbf {E}}}_{\text {ref}} ({{\textbf {r}}},k)\, d{{\textbf {r}}}. \end{aligned}$$

where ${{\textbf {E}}}_{\text {scat}}$ and ${{\textbf {E}}}_{\text {ref}}$ are the time-harmonic fields within the sample and reference arms, respectively. We assume an $\exp ({-i\omega t})$ time dependence, where $\omega = kc$ and *c* being the freespace speed of light. Here, each integral is evaluated on the planes $\Omega _{b}$ and $\Omega _{\text {ref}}$, and $\varvec{\phi }({{\textbf {r}}},k) = [\phi _{x}({{\textbf {r}}},k),\;\phi _{y}({{\textbf {r}}},k),\; 0\,]^{\text {T}}$ is the fiber mode imaged into each plane, where we assume this mode is purely transverse to the *z*-direction. As OCT systems typically involve illumination and collection of light on the same side of the sample (known as reflection, or backscattering mode), we will primarily be concerned with this scenario, where the planes $\Omega _{b}$ and $\Omega _{\text {ref}}$ are positioned on the same side of the sample/mirror as the illuminating source, as illustrated in Fig. 1a.

To be explicit, in this paper we will define the inverse problem in OCT to mean the use of the measured detector signal defined in Eq. 1, or a collection of such measurements, to determine the distribution of the optical parameters within the sample. In the remainder of this paper we will primarily be concerned with the recovery of the refractive index distribution ($n=\sqrt{\epsilon _{r}}$) defined as the square root of the relative permittivity.

### Scattered field inverse problem

A more general setting than the OCT inverse problem described above, is to assume we have access to the full scattered field, ${{\textbf {E}}}_{\text {scat}}$ , on a plane in the sample region, rather than only the detector signal $I_{\text {det}}$. Whilst ${{\textbf {E}}}_{\text {scat}}$ is not measurable in typical OCT systems, it is possible to determine it in some specific experimental setups^11,12^. We will refer to the problem of recovering refractive index from ${{\textbf {E}}}_{\text {scat}}$ as the scattered field inverse problem. This scattered field inverse problem is of interest as it presents an upper bound, or “best-case” outcome of the OCT inverse problem, although it is still severely ill-posed as we will demonstrate. Crucially relevant for our purposes, any determination made about the non-uniqueness of the scattered field inverse problem also informs us about the non-uniqueness of the OCT inverse problem.

**Figure 2:**
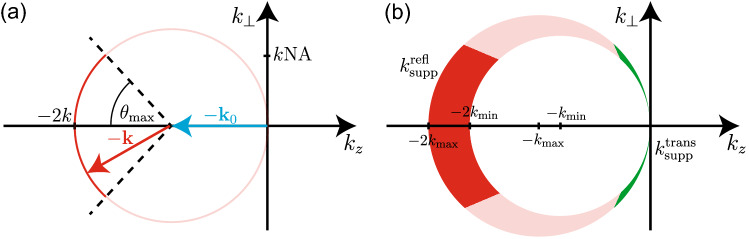
(**a**) Diagram showing the recoverable spatial frequencies (dark red section of circle) of the sample’s scattering potential for the backscattering configuration with plane wave illumination and detection on the plane $\Omega _{b}$ subtended by angle $\theta _{\text {max}}$. (**b**) Recoverable spatial frequencies for broadband illumination with wavenumbers spanning from $k_{\text {min}}$ to $k_{\text {max}}$, shown as dark red section of the crescent region, $k^{\text {refl}}_{\text {supp}}$. Also shown in green are the recoverable spatial frequencies for an alternate scenario where measurements are instead made on the forward plane, $\Omega _{f}$, where this region is denoted by $k^{\text {trans}}_{\text {supp}}$.

The scattered field inverse problem has been thoroughly studied in the field of optical diffraction tomography, which we will briefly review here. We first define the scattering potential of a sample as:

3 $$\begin{aligned} F({{\textbf {r}}}) = -k^{2} \left( m^{2}({{\textbf {r}}}) -1\right) \;, \end{aligned}$$

where *k* is the freespace wavenumber of the illuminating wave, and $m = n_{s}/n_{b}$ is the refractive index contrast, with $n_{s}$ representing the sample refractive index, and $n_{b}$ representing the background refractive index. Referring to Fig. 1b which shows the general setup for the scattered field inverse problem (diffraction tomography problem), we consider an incident plane wave source with wavevector ${{\textbf {k}}}_{0} = k\hat{{{\textbf {s}}}}_{0}$ which illuminates the scattering object. We define the measurement plane, $\Omega _{b}$, as the plane normal to the *z*-axis positioned between the source and the object at $z = z_{b}$, with the object at the origin. Note that the figure is projected on to the *x*-*z* plane for simplicity. Under a weak scattering approximation where we assume objects have only a small refractive index contrast (i.e., under the first-order Born approximation), the relationship between the Fourier transform of the scattering potential, and the Fourier transform of the scattered field with respect to the *x* and *y* directions on the measurement plane is given by^13^,

4 $$\begin{aligned} {\tilde{F}}_{\textrm{Born}}({{\textbf {K}}}) = \frac{ik_{z}}{\pi }\text {exp}\left( i k_{z} z_{b} \right) {\tilde{E}}_{\text {scat}}(k_{x},k_{y};z_{b}), \end{aligned}$$

where we have ${{\textbf {k}}} = k\hat{{{\textbf {s}}}} = (k_{x},k_{y},k_{z})$, and where

5 $$\begin{aligned} {{\textbf {K}}} = {{\textbf {k}}} - {{\textbf {k}}}_{0}. \end{aligned}$$

We note that in Eq. 4 we have assumed a scalar description of the electric field as with the original derivation in Ref. ^13^, although more general polarization sensitive formulations are possible for scenarios with non-isotropic media^14^. Additionally, Eq. 4 is only valid under the condition $k_{x}^2 + k_{y}^2 \le k^2$, i.e., excluding information carried by evanescent plane wave components of the scattered field. Equation 4 indicates that under the approximation of a weakly scattering object, we can directly infer the Fourier transform of the scattering potential at each spatial frequency ${{\textbf {K}}}$, by examining the Fourier transform of the measured field on the plane $\Omega _{b}$. This however also demonstrates that for a limited range of illumination and detection directions, there is a fundamental limit to the observable spatial frequencies of the scattering potential. For instance, if we consider only a single plane wave illumination direction with ${{\textbf {k}}}_{0} = k\hat{{{\textbf {z}}}}$, and assume we have access to only a portion of the infinite plane at $z_{b}$ subtended by an angle $\theta _{\text {max}}$ from the center of the object, which characterizes the detection Numerical Aperture, NA $=n_{b}\,\text {sin}\,\theta _{\textup{max}}$, then we have the limitation $\sqrt{k_{x}^2 + k_{y}^2} \le k\text {NA}$. Thus, the range of recoverable spatial frequencies of the scattering potential occupies a section of a spherical shell of radius *k*, centered at $(0,0,-k)$. In Fig. 2a, this region is shown as a red arc in a projected plane, $k_{\perp }$, perpendicular to $k_{z}$. For a detection region on the measurement plane covering a small NA, the observable spatial frequencies are only those in close vicinity to $(0,0,-2k)$; in particular this means that none of the low spatial frequency information of the scattering potential is available in the $k_{z}$-direction. When using broadband illumination spanning from $k_{\text {min}}$ to $k_{\text {max}}$, such as in Fourier domain OCT, the range of observable spatial frequencies increases^15,16^. This is visualized in Fig. 2b as the dark red section of a crescent shape in the $k_{\perp }$-$\,k_{z}$ plane. Note however, in alternative source-detector geometries, such as a transmission geometry with the same NA (where measurements are made on the plane $\Omega _{f}$ shown in Fig. 1b), an entirely separate range of spatial frequencies of the object are observable, and are in the low frequency region. This is shown for the broadband case in Fig. 2b as the green region of the crescent. In the following we will refer to these regions/supports, as $k_{\textrm{supp}}^{\textrm{refl}}$ and $k_{\textrm{supp}}^{\textrm{trans}}$ respectively.

A comprehensive description of related imaging modalities and their associated transfer functions can be found in references^16–18^. However, with this simple diffraction tomography description of the scattered field inverse problem, there clearly exists a fundamental restriction on the amount of information about the scattering potential (and thus the refractive index) that we can recover in typical OCT geometries, with illumination and detection directions being almost anti-parallel. In situations where the sample is able to be rotated with freedom, with the source-detection configuration remaining static, additional information is available^19^. However even in this scenario the observable spatial frequencies are still restricted to those with magnitudes $\vert {{\textbf {K}}} \vert \approx 2k$, with low spatial frequency information remaining unattainable. A natural question arises, does this restriction still apply to samples which violate the weak scattering approximation? In other words, does the same “missing frequency problem” apply when the incident field can no longer be assumed to be unperturbed by the sample itself, such as in the case with physically large and/or high contrast samples? Answering this question first requires a means of solving the full non-linear inverse problem, which we present in the next section. This method then allows us to investigate the solution of such scattering scenarios in the results section.

## Methods

### Iterative inversion algorithm

In this section we employ an alternative approach to the scattered field inverse problem discussed above. Here, rather than operating under the weak-scattering limit, which allows for an approximate analytic solution, we instead cast the scattered field inverse problem as an optimization problem. Using this approach, no assumptions regarding the scale of the scattering potential are necessary (i.e. no first-order Born approximation is necessary). The metric we wish to minimize in this approach is the difference between the true scattered electromagnetic field due to the sample, defined by ${{\textbf {E}}}^{\text {true}}_{\text {scat}}$ , ${{\textbf {H}}}^{\text {true}}_{\text {scat}}$ , and the scattered electromagnetic field, ${{\textbf {E}}}_{\text {scat}}$ , ${{\textbf {H}}}_{\text {scat}}$ generated by some estimation of the sample scattering potential. In the above sections we were concerned with the time-harmonic fields resulting from continuous-wave broadband illumination. Here, however, in order to compute the fields arising from an estimation of the scattering potential, we are required to utilize time-domain simulations with a temporally pulsed illumination source. This temporal illumination pulse is derived such that the desired spectral bandwidth is achieved, and is introduced into the simulations as a time varying magnetic current density^20^. The spectral bandwidth profile we target is a Gaussian profile with a Full-Width at Half-Maximum (FWHM) of 100 nm, and a central wavelength of 1300 nm. Whilst in practice the fields ${{\textbf {E}}}^{\text {true}}_{\text {scat}}$ and ${{\textbf {H}}}^{\text {true}}_{\text {scat}}$ would be determined experimentally for each wavenumber, and transformed into a time-domain representation to be compared to the simulated fields ${{\textbf {E}}}_{\text {scat}}$ and ${{\textbf {H}}}_{\text {scat}}$ , in this study we will be entirely simulating the example problems, and will thus begin with simulated versions of the true scattered fields in the time domain as well. We will use the L2 norm of the difference between these fields integrated over the time interval [0, *T*], and over the measurement plane $\Omega _{b}$, given as:

6 $$\begin{aligned} \begin{aligned} L({{\textbf {p}}},{{{\textbf {E}}}_{\text {scat}}},{{{\textbf {H}}}_{\text {scat}}})&= \frac{1}{2} \int _{0}^{T}\int _{\Omega _{b}} ( \left\| {{\textbf {E}}}_{\text {scat}} ({{\textbf {r}}},{{\textbf {p}}},t) - {{\textbf {E}}}_{\text {scat}} ^{\text {true}}({{\textbf {r}}},t) \right\| ^{2}\\&\quad +n_{0}^{2}\left\| {{\textbf {H}}}_{\text {scat}} ({{\textbf {r}}},{{\textbf {p}}},t) - {{\textbf {H}}}_{\text {scat}} ^{\text {true}}({{\textbf {r}}},t) \right\| ^{2} ) \,d{{\textbf {r}}}\,dt\, \end{aligned} \end{aligned}$$

where ${{\textbf {p}}} = [\epsilon ({{\textbf {r}}}),\rho ({{\textbf {r}}}),\mu ({{\textbf {r}}})]^{\text {T}}$ is an optical parameter vector describing the permittivity, conductivity and permeability of the estimated sample, respectively. To compute the electromagnetic fields in our simulated example problems, we use a Psuedo-Spectral Time-Domain (PSTD) model of pulsed electric field propagation^10^. To find the internal optical parameters, ${{\textbf {p}}}$, from time-resolved measurements made at the plane, $\Omega _{b}$, we minimize the cost metric via a process of iterative gradient descent. We follow a process outlined in Ref.^21^, which uses the method of Lagrange multipliers for this constrained optimization problem. The process starts by forming an augmented cost metric, $L^{a}$, which includes the constraint that our electromagnetic field must satisfy Maxwell’s equations,

7 $$\begin{aligned} \begin{aligned} L^{a}({{\textbf {p}}},{{\textbf {E}}}_{\text {scat}} ,{{\textbf {H}}}_{\text {scat}} )&= L({{\textbf {p}}},{{\textbf {E}}}_{\text {scat}} ,{{\textbf {H}}}_{\text {scat}} )\\&\quad + \int _{0}^{T}\int _{V_{\textrm{samp}}} \left[ {\textbf {e}}_{\text {adj}}\cdot \left( \nabla \times {{\textbf {H}}}_{\text {samp}} - \epsilon \, \frac{\partial {{\textbf {E}}}_{\text {samp}}}{\partial t} - \sigma {{\textbf {E}}}_{\text {samp}} - {{\textbf {J}}}\right) \right. \\&\quad \left. + {{\textbf {h}}}_{\text {adj}}\cdot \left( \nabla \times {{\textbf {E}}}_{\text {samp}} + \mu \, \frac{\partial {{\textbf {H}}}_{\text {samp}}}{\partial t}\right) \right] d{{\textbf {r}}}dt \end{aligned} \end{aligned}$$

where the integration is carried out over the volume in the sample region $V_{\textrm{samp}}$. In the above, ${\textbf {e}}_{\text {adj}}$ and ${{\textbf {h}}}_{\text {adj}}$ are Lagrange multipliers, ${{\textbf {J}}}$ is the source current density, and we point out that the fields ${{\textbf {E}}}_{\textrm{samp}}$, ${{\textbf {H}}}_{\textrm{samp}}$ are the total fields in the sample region (including source field and scattered field). The Lagrange multipliers themselves satisfy Maxwell’s Equations, and can be computed by backpropagating the residual electric field, described by

8 $$\begin{aligned} {{\textbf {E}}}_{\textrm{res}} = \left( {{\textbf {E}}}_{\text {scat}} - {{\textbf {E}}}_{\text {scat}} ^{\text {true}} \right) , \end{aligned}$$

into the medium from the measurement plane, $\Omega _{b}$^21^. We introduce the residual electric field source into the PSTD algorithm using a source condition that generates the magnetic field from knowledge of the electric field^20^. This results in what is referred to as an internal adjoint field described by ${\textbf {e}}_{\rm adj}$, ${{\textbf {h}}}_{\text {adj}}$ that propagates from the measurement plane, into the medium. In order to compute a gradient of the cost metric *L* with respect to the optical parameters ${{\textbf {p}}}$, we require the scattered field ${{\textbf {E}}}_{\text {scat}}$, ${{\textbf {H}}}_{\text {scat}}$ and the adjoint field, ${{\textbf {e}}}_{\text {adj}}$, ${{\textbf {h}}}_{\rm adj}$ described at all locations within the sample over the time range [0, *T*]. Fortunately, both of these fields can be simulated with the same PSTD solver. This is the main benefit of using this “forward-adjoint” method. For the purposes of this study, we will assume we are dealing with non-magnetic materials i.e., $\mu = \mu _{0}$, and we will also assume non-conducting and non-dispersive optical properties. This reduces the problem to finding only the dielectric permittivity, $\epsilon ({{\textbf {r}}})$, or equivalently the scattering potential, $F({{\textbf {r}}})$. The gradient of the augmented cost function with respect to the permittivity is described in Ref.^21^ as:

9 $$\begin{aligned} \frac{\partial L^{a}}{\partial \epsilon } = - \int _{0}^{T} {{\textbf {e}}}_{\text {adj}} \cdot \frac{\partial {{\textbf {E}}}_{\text {scat}} }{\partial t}\, dt\;. \end{aligned}$$

Once computed, the gradient described in Eq. 9 can then be used in iterative gradient-based optimization techniques to approximate the internal permittivity (and thus the scattering potential, and refractive index). In this work, we use the Polak-Ribière update method, as described in Ref.^21^. To summarize the above description, the iterative inversion algorithm is presented in Algorithm 1. Within this algorithm, the PSTD solver is represented as $PSTD\left\{ F,S \right\}$, where the first argument is the scattering potential of the sample, *F*, and the second argument is the source field, *S*. For the forward field, $S_{\textrm{pw}}$ represents an *x*-polarised plane wave pulse travelling in the $+z$-direction, with central wavelength $\lambda _{c}$. For the adjoint field, $S_{\textrm{res}}({{\textbf {E}}}_{\textrm{res}})$ is a function that generates the residual source condition from the field given in Eq. 8. The parameters used in the PSTD solver are provided in Table 1. The Polak-Ribière update is a function of both the current gradient term, and previous update term^22^, and is denoted by *PR* in Algorithm 1. Lastly, for the remainder of this paper, we will use superscripts “true” to represent the true object, “linrec” to represent the linear reconstruction of the object predicted by diffraction tomography theory, and “itrec” to represent the reconstruction of the sample returned by the iterative solver.
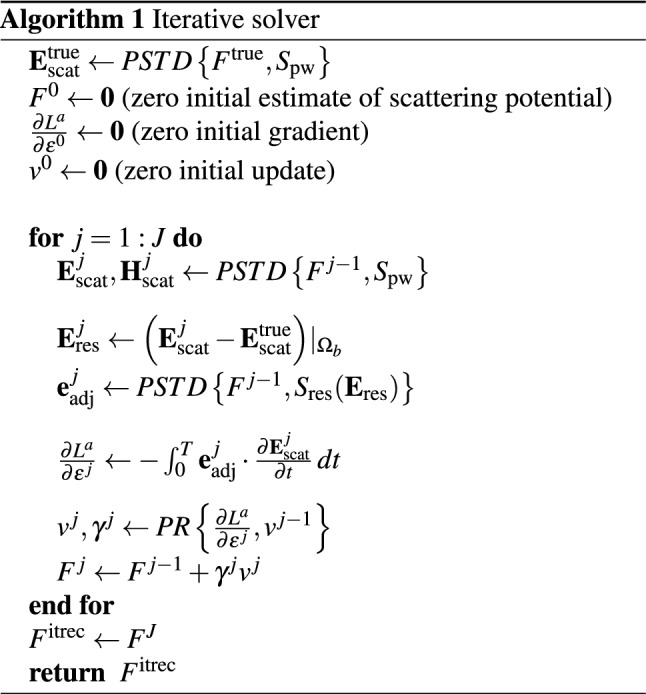

**Table 1:** Simulation parameters used in the Psuedo spectral time domain (PSTD) solver.

Parameter	Value
Central wavelength, $\lambda _{c}$	$1300\,$nm
Temporal pulse width (FWHM)	$7.46 \times 10^{-14}$s
Spectral bandwidth^a^	$100\,$nm (FWHM)
Grid cell size	$\lambda _{c}/12$
Domain size	$200 \times 200$ grid cells
PML size	20 cells
Timestep size	$1.26 \times 10^{-16}$ s
Total timesteps	4000

^a^Equivalent spectral bandwidth derived from temporal width of illumination pulse.

## Results

In this section we investigate the performance of the iterative solver, and compare the results to what would be expected from diffraction tomography theory, as discussed above. Whilst the PSTD solver used in the iterative algorithm is capable of full three dimensional modelling, in this study we have enforced that all media are uniform along the *y*-dimension, thus reducing the problem to a two-dimensional scenario for ease of illustration. In the following, we will investigate three different types of sample object as described in Table 2.

**Figure 3:**
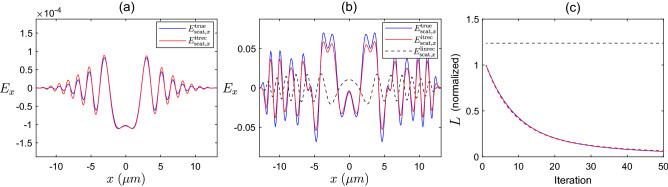
(**a**) *x*-component of the time-dependent scattered field measured on $\Omega _{b}$ for the true point-like sample, $F^{\textrm{true}}_{1}$ (blue), and for the iterative solver reconstruction, $F^{\textrm{itrec}}_{1}$ (red) at timestep 1620 of the PSTD simulation. (**b**) *x*-component of the time-dependent scattered field measured on $\Omega _{b}$ for the true high contrast disk sample, $F^{\textrm{true}}_{3}$ (blue), for the iterative solver reconstruction, $F^{\textrm{itrec}}_{3}$ (red), and for the linear reconstruction (black dashed) at timestep 2070 of the PSTD simulation. (**c**) cost metric, *L*, normalized to the initial value (with an empty estimate for the scattering potential) over 50 iterations of the iterative solver for the point scatterer (blue), and for the high constrast disk (red dashed). Also shown is the normalized cost metric value for the linear reconstructed sample (black dashed).

**Figure 4:**
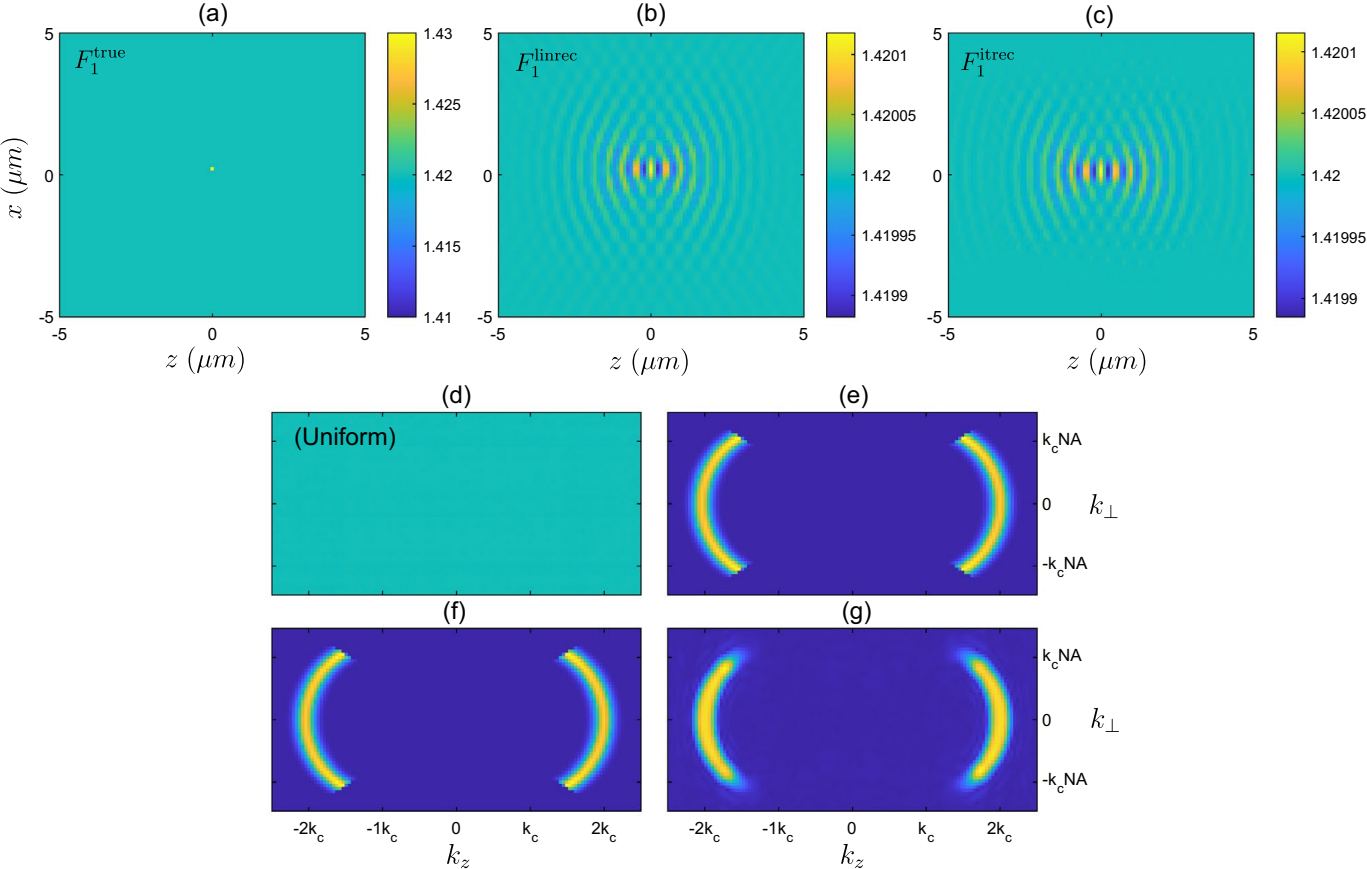
(**a**) Refractive index corresponding to the scattering potential of the point-like sample $F_{1}$. (**b**) Refractive index corresponding to the linear reconstruction $F^{\textrm{linrec}}_{1}$. (**c**) Refractive index corresponding to the iterative solver reconstruction $F^{\textrm{itrec}}_{1}$. (**d**) Magnitude of Fourier transform, $\vert {\tilde{F}}^{\textrm{true}}_{1}({{\textbf {k}}})\vert$, of the true sample (which in this case is simply uniform). (**e**) Theoretical *k*-space filter, $f_{\Omega _{b}}({{\textbf {k}}})$. (**f**) Magnitude of Fourier transform of true sample after being filtered $\vert {\tilde{F}}^{\textrm{true}}_{1}({{\textbf {k}}}) \times f_{\Omega _{b}}({{\textbf {k}}})\vert$, i.e. (**d**) multiplied by (**e**). (**g**) Magnitude of the Fourier transform of the reconstructed sample provided by the iterative solver, $\vert {\tilde{F}}^{\textrm{itrec}}_{1}({{\textbf {k}}})\vert$.

**Table 2:** Properties of each of the three samples investigated.

Name	Sample description	$n_b$	$n_s$	Diameter
$F_1$	Low contrast point	1.42	1.43	$\lambda _{c}$
$F_2$	Low contrast disk	1.42	1.43	$3\lambda _{c}$
$F_3$	High contrast disk	1.42	1.82	$3\lambda _{c}$

### Reflection geometry

In our first example, we attempt to reconstruct the refractive index distribution of the low contrast point-like sample, $F_{1}$. This sample is shown in Fig. 4a, and consists of only one grid cell of size $\lambda _{c}/12$ within the *x*-*z* plane (as mentioned above this technically extends infinitely in the *y*-dimension). After performing 50 steps of the iterative solver, the simulated field ${{\textbf {E}}}_{\text {scat}} ^{\text {itrec}}$ scattered by the reconstructed object, $F^{\textrm{itrec}}_{1}$, closely resembles that of the field, ${{\textbf {E}}}^{\text {true}}_{\textrm{scat}}$, scattered from the true object, $F^{\textrm{true}}_{1}$. These two fields are compared at a particular timestep of each PSTD simulation in Fig. 3a. Also shown in Fig. 3c is the reduction of the cost metric, *L*, over the course of 50 iterations of the algorithm, where the cost metric has been normalized to the initial value when a zero-valued scattering potential is present in the simulation. The object reconstructed by the iterative solver, $F^{\textrm{itrec}}_{1}$ is shown in Fig. 4c. It is immediately clear that the reconstructed object has refractive index variations covering a much larger area than the true point-like object. Secondly, the maximum refractive index contrast of the reconstructed object is much less than the contrast of the true scattering object. Another notable characteristic is the oscillating pattern of the refractive index along the *z*-direction, which has a length scale of $\approx \lambda _{c}/2$, or , equivalently, a spatial frequency of $\approx 2k_{c}$. In Fig. 4b we show the linear reconstruction that we would expect to see from diffraction tomography theory, $F^{\textrm{linrec}}_{1}$. This linear reconstruction is found by filtering the true object in *k*-space with the theoretical filter derived from diffraction tomography theory for measurements made on the plane $\Omega _{b}$, where the filter is denoted by $f_{\Omega _{b}}$ (filter shown in Fig. 4e). This filter occupies the support illustrated in Fig. 2, (i.e., $\textrm{supp}\left[ f_{\Omega _{b}}\right] = k^{\text {refl}}_{\text {supp}}$), and has a Gaussian weighting over contributions from $k_{\textrm{min}}$ to $k_{\textrm{max}}$, with central wavenumber $k_{c}$, describing the equivalent spectral profile of the temporally pulsed source used in the PSTD simulations. This linear reconstruction, $F^{\textrm{linrec}}_{1}$, agrees closely with the reconstruction provided by the iterative solver, even given the notable differences between the two approaches. The shape seen in Fig. 4b is easy to interpret because the filter in Fig. 4e is both bandlimited (giving rise to the oscillations) and limited-angle (giving rise to the angular variation).

In Fig. 4d, we show the magnitude of the Fourier transform of the true object, $\vert {\tilde{F}}^{\textrm{true}}_{1}\vert$, which in the case of this point-like object is uniform. Fig. 4e shows the theoretical filter $f_{\Omega _{b}}$. Figure 4f shows the product of the true object and the filter, $\vert {\tilde{F}}^{\textrm{true}}_{1}({{\textbf {k}}}) \times f_{\Omega _{b}}({{\textbf {k}}})\vert$. And finally, in Fig. 4g we show the magnitude of the Fourier transform of the iterative solver reconstruction, $\vert {\tilde{F}}^{\textrm{itrec}}_{1}({{\textbf {k}}})\vert$. It is clear from this comparison that the reconstruction provided by the iterative solver occupies approximately the same support, $k^{\text {refl}}_{\text {supp}}$ as predicted by diffraction tomography theory, with some minor differences in the weighting of the spatial frequencies, particularly around the edges of the support.

**Figure 5:**
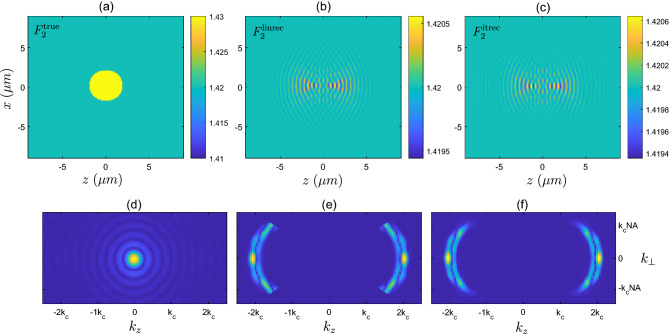
(**a**) Refractive index corresponding to the scattering potential of the low contrast disk sample $F_{2}$. (**b**) Refractive index corresponding to the linear reconstruction $F^{\textrm{linrec}}_{2}$. (**c**) Refractive index corresponding to the iterative solver reconstruction $F^{\textrm{itrec}}_{2}$. (**d**) Magnitude of Fourier transform, $\vert {\tilde{F}}^{\textrm{true}}_{2}({{\textbf {k}}})\vert$, of the true low contrast disk sample. (**e**) Magnitude of Fourier transform of true sample after being filtered $\vert {\tilde{F}}^{\textrm{true}}_{2}({{\textbf {k}}}) \times f_{\Omega _{b}}({{\textbf {k}}})\vert$. (**f**) Magnitude of the Fourier transform of the reconstructed sample provided by the iterative solver, $\vert {\tilde{F}}^{\textrm{itrec}}_{2}({{\textbf {k}}})\vert$.

**Figure 6:**
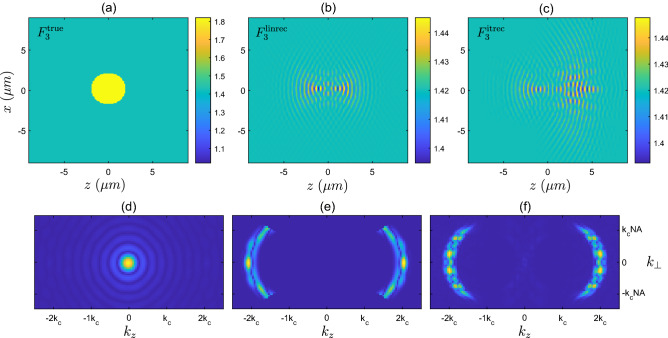
(**a**) Refractive index corresponding to the scattering potential of the high contrast disk sample $F_{3}$. (**b**) Refractive index corresponding to the linear reconstruction $F^{\textrm{linrec}}_{3}$. (**c**) Refractive index corresponding to the iterative solver reconstruction $F^{\textrm{itrec}}_{3}$. (**d**) Magnitude of Fourier transform, $\vert {\tilde{F}}^{\textrm{true}}_{3}({{\textbf {k}}})\vert$, of the true high contrast disk sample. (**e**) Magnitude of Fourier transform of true sample after being filtered $\vert {\tilde{F}}^{\textrm{true}}_{3}({{\textbf {k}}}) \times f_{\Omega _{b}}({{\textbf {k}}})\vert$. (f) Magnitude of Fourier transform of the reconstructed sample provided by the iterative solver, $\vert {\tilde{F}}^{\textrm{itrec}}_{3}({{\textbf {k}}})\vert$.

For the next two examples, we perform a similar comparison using the larger disk-shaped scattering objects, $F_{2}$ and $F_{3}$, with low and high refractive index contrasts, respectively. In Fig. 5a–c we display the refractive index of the low contrast disk sample $F_{2}$, as well as both the linear reconstruction $F^{\textrm{linrec}}_{2}$, and the reconstruction provided by the iterative solver after 50 iterations, $F^{\textrm{itrec}}_{2}$. Again we can observe that both reconstructions closely agree with each other, yet differ significantly from the true object. The dominant feature of both reconstructions is the $2k_{c}$ spatial frequency along the *z*-direction. It is also observed that both reconstructions are symmetric along the *z*-direction about the center of the object. Although not shown explicitly, we note that the scattered fields produced by all three objects in Fig. 5a–c show close agreement, similar to the agreement shown in Fig. 3a. In Fig. 5d–f we again show the process of applying the filter $f_{\Omega _{b}}$ to the Fourier transform of the true object $\vert {\tilde{F}}^{\textrm{true}}_{2}\vert$, as well as showing the Fourier transform of the iterative solver reconstruction $\vert {\tilde{F}}^{\textrm{itrec}}_{2}\vert$.

The high contrast disk object, $F_{3}$, shows a notably different reconstruction from the iterative solver in Fig. 6c, when compared to the linear reconstruction (b). With this sample, we expect that the weak scattering approximation is violated, and therefore that the linear reconstruction will no longer produce the correct scattered field, nor will it agree with the sample reconstruction provided by the iterative solver. The scattered fields are compared in Fig. 3b, where the iterative reconstruction closely resembles the true field, whilst the linear reconstruction produces an inaccurate scattered field, as expected. Also shown in Fig. 3c are the cost metric values as a function of the number of iterations of the solver, along with the cost metric value associated with the linear reconstruction. Looking more closely at Fig. 6 where the different reconstructions are compared, the iterative solver reconstruction is distributed over a larger area, and is also asymmetric along the *z*-direction. Interestingly, the left hand side of the $F^{\textrm{itrec}}_{3}$ appears to agree more closely with $F^{\textrm{linrec}}_{3}$, which due to the illumination travelling in the $+z$ direction (coming from the left hand side of the domain) suggests that the early arriving portion of the scattered field pulse is somewhat similar to the lower contrast disk (to within a constant). However, as the right hand side of the object is responsible for the later arriving portion of the scattered field pulse, it appears that the higher contrast leads to this field being significantly different from the low contrast case. It is also interesting to analyse the reconstruction in the Fourier domain (Fig. 6d–f), where it is seen that the support of the reconstructed object appears largely the same and in approximate agreement with the predicted $k^{\text {refl}}_{\text {supp}}$. Within this region of support, however, there is a significantly different weighting of the spatial frequencies compared to the low contrast cylinder of the same shape, suggesting some additional interference between spatial frequency components. Additionally on close inspection, there appear to be some spatial frequencies outside of the support $k^{\text {refl}}_{\text {supp}}$ predicted by diffraction tomography, particularly in the region of low frequencies, although these are of low magnitude (barely visible). Whilst the appearance of these spatial frequency components of the reconstruction are not entirely understood, they are potentially due to strong scattering by the high contrast object generating effective additional illumination directions. Whilst acknowledging that we must be careful not to overgeneralize the diffraction tomography description of observable frequencies, and their relationship to directions of illumination and detection (as the underlying assumptions are violated in this scenario), the appearance of these low frequency components suggests that the solution in the strong scattering scenario may differ with regards to its support in spatial frequency space. Nevertheless, in this example, at least, the dominant spatial frequencies remain those occupying the region described by $k^{\text {refl}}_{\text {supp}}$ .

### Alternate geometries

**Figure 7:**
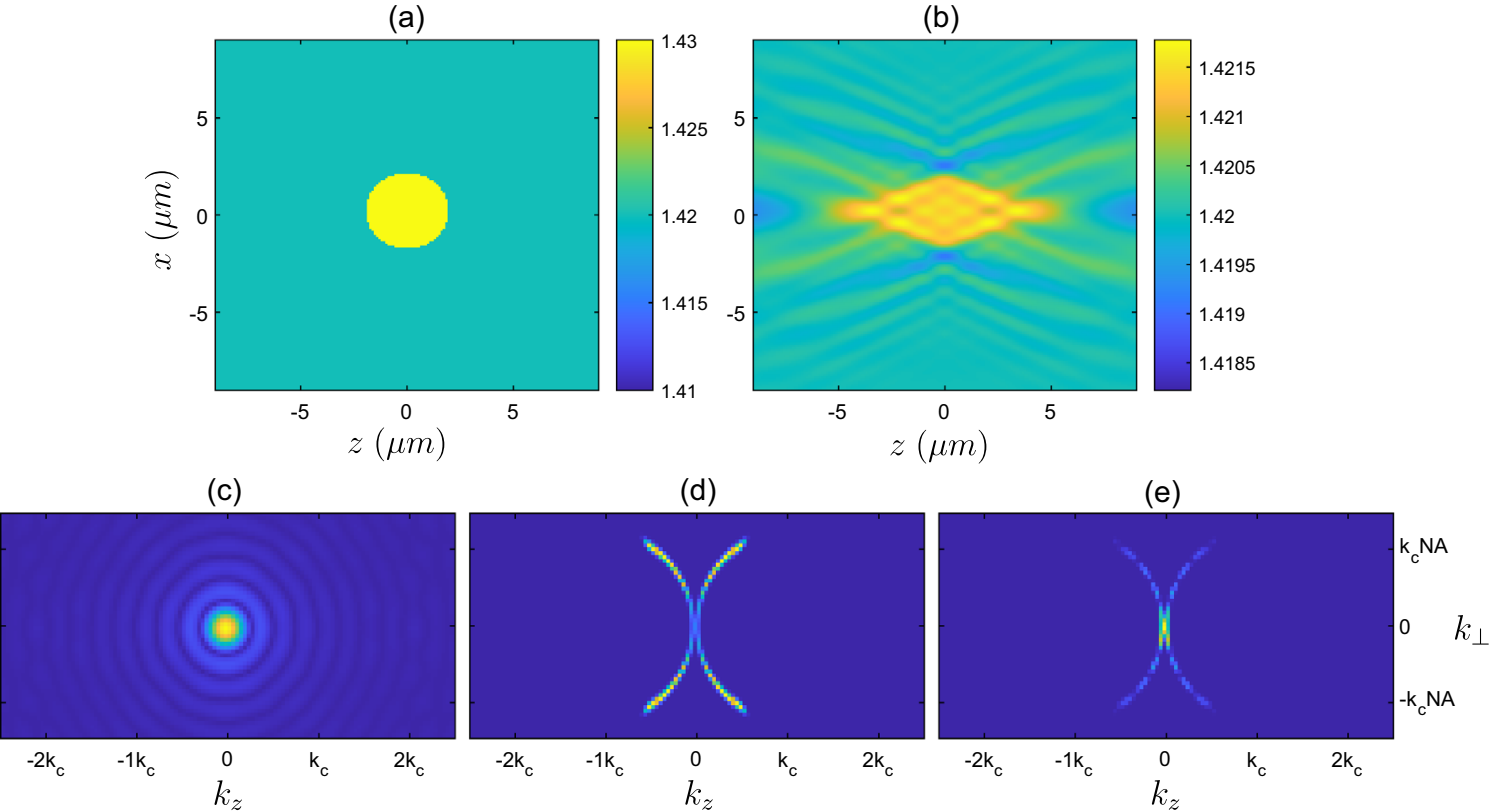
(**a**) Refractive index corresponding to the scattering potential of the low contrast cylinder $F_{2}$. (**b**) Refractive index corresponding to the linear reconstruction $F^{\textrm{linrec}}_{2}$ when measuring using the forward plane, $f_{\Omega _{f}}$. (**c**) Magnitude of Fourier transform, $\vert {\tilde{F}}^{\textrm{true}}_{2}({{\textbf {k}}})\vert$, of the true low contrast disk sample. (**d**) Theoretical *k*-space filter, $f_{\Omega _{f}}({{\textbf {k}}})$ for the forward measurement plane. (**e**) Magnitude of Fourier transform of true sample after being filtered $\vert {\tilde{F}}^{\textrm{true}}_{5}({{\textbf {k}}}) \times f_{\Omega _{f}}({{\textbf {k}}})\vert$, i.e. (**c**) multiplied by (**d**).

We now investigate some sample reconstructions that are expected when using source-detector geometries that differ from the reflection geometry. Although the geometries we present here are not typically relevant to OCT (with some exceptions, e.g. Ref.^23^), these highlight the differences in object reconstruction when low spatial frequency information is available, rather than just spatial frequencies with magnitudes of $\approx 2k_{c}$. In Fig. 7 we compare the true low contrast disk sample ($F_{2}$) and the linear reconstruction for the scenario where measurements are made on the forward plane only (on the plane $\Omega _{f}$ as pictured in Fig. 1a), with plane wave illumination incident in the $+z$ direction. The reconstructed object shown in Fig. 7b is clearly very different from the reconstruction in the backscattering case for the same object shown in Fig. 5b. Figure 7c–e illustrates the scenario in *k*-space, with the filter used in the forward detection geometry, $f_{\Omega _{f}}({{\textbf {k}}})$, occupying the support $k^{\text {trans}}_{\text {supp}}$ as shown in Fig. 2b.

**Figure 8:**
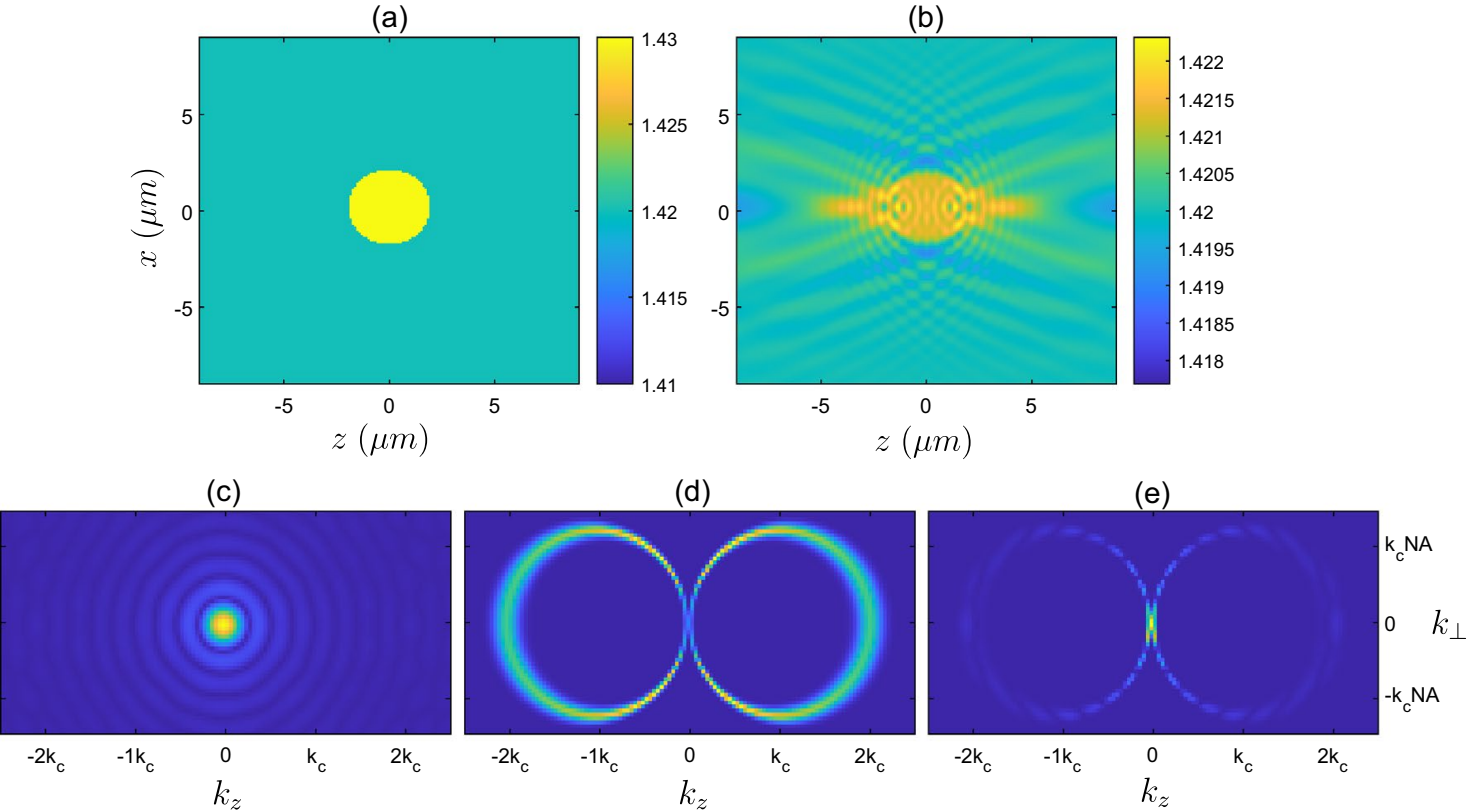
(**a**) Refractive index corresponding to the scattering potential of the low contrast cylinder $F_{2}$. (**b**) Refractive index corresponding to the linear reconstruction $F^{\textrm{linrec}}_{2}$ when measuring using all detection directions (with plane wave illumination in the $+z$-direction only). (**c**) Magnitude of Fourier transform, $\vert {\tilde{F}}^{\textrm{true}}_{2}({{\textbf {k}}})\vert$, of the true low contrast disk sample. (**d**) Theoretical *k*-space filter for the detection over all angles, $f_{\Omega _{\text {all}}}({{\textbf {k}}})$. (**e**) Magnitude of Fourier transform of true sample after being filtered $\vert {\tilde{F}}^{\textrm{true}}_{2}({{\textbf {k}}}) \times f_{\Omega _{\text {all}}}({{\textbf {k}}})\vert$, i.e. (**c**) multiplied by (**d**).

In Fig. 8 we show the scenario where the scattered field is able to be measured in all directions, and again with plane wave illumination in the *z*-direction. Specifically, this scenario would require measurement of the field on a series of planes surrounding the sample. Here, the theoretical filter $f_{\Omega _{\text {all}}}$ occupies the entire crescent region shown in Fig. 2b. In this case, the reconstructed sample shown in Fig. 8b begins to resemble the true object in Fig. 8a, and can also visually be seen to contain both low and high spatial frequencies.

## Conclusions

In this study we have demonstrated that attempting to reconstruct sample refractive index using back scattered electromagnetic field data results in missing spatial frequencies of the sample, particulary low spatial frequencies along the axial direction. Whilst this “missing frequency problem” is expected^13^, we demonstrated that maps of scattering potential reconstructed using a non-linear iterative technique are approximately equivalent to the linear reconstructions obtained using diffraction tomography theory in the weak scattering limit. These reconstructions are characterized by a limited support in spatial frequency space, $k^{\text {refl}}_{\text {supp}}$. For higher contrast scattering samples, the scattering potentials obtained using the linear reconstruction (based on diffraction tomography theory) fail to replicate the true scattered field of the object. However, the non-linear iterative solver results in a scattering potential map that produces a scattered field closely resembling the true field. These reconstructions however were still characterized in spatial frequency space by a dominant range of frequencies, closely related to the support predicted by diffraction tomography theory $k^{\text {refl}}_{\text {supp}}$. However, some low spatial frequencies were present outside of this region (but with very low magnitude).

The purpose of our investigation of the scattered field inverse problem was to investigate the limitations inherent to the OCT inverse problem. We demonstrated that in both the weak and strong scattering limits, the scattered field inverse problem in the reflection geometry is ill-posed, as our solutions demonstrated non-uniqueness. In particular we showed that incorrect scattering potentials are retrieved by our non-linear iterative technique which still produce scattered fields which closely match the true scattered fields. Given that the OCT inverse problem is further limited compared to the scattered field inverse problem, it is clear that the OCT inverse problem is also ill-posed. In other words, if the scattered electric fields from two different objects are equivalent on the plane $\Omega _{b}$, then any signals derived from these fields will also be equivalent, such as an OCT signal defined via Eqs. 1 and 2. Furthermore, we demonstrate that OCT is only able to access relatively limited regions of the sample spatial frequency spectrum support using a conventional reflection geometry.

In order to reduce the ill-posedness of the problem without making limiting assumptions, additional illumination or viewing angles are required. Such mechanisms for modifying the incident beam angle in OCT have previously been demonstrated^24,25^, although these examples would only marginally improve the support $k^{\text {refl}}_{\text {supp}}$ . Additionally, although not explored here, modifications to the optimization process can also be made which may alter the reconstructions. These modifications could include regularization (such as total variation regularization^26^), or other means of making use of prior information in the solution.

## Data Availability

The data that support the findings of this study are available from the corresponding author upon reasonable request.
